# Carbon-Nanotube-Enabled Low-Threshold Laser Lift-Off for Ultra-Thin Polyimide Films

**DOI:** 10.3390/nano16090527

**Published:** 2026-04-27

**Authors:** Junwei Fu, Yachong Xu, Run Bai, Zhenzhen Sun, Yili Zhang, Rui Yang, Zijuan Han, Fanfan Wang, Boyuan Cai

**Affiliations:** 1 School of Artificial Intelligence Science and Technology, University of Shanghai for Science and Technology, Shanghai 200093, China; 2 Institute of Photonic Chips, University of Shanghai for Science and Technology, Shanghai 200093, China; 3 Shenzhen Han’s Semiconductor Equipment Technology Co., Ltd., Shenzhen 518101, China; 4 School of Mechatronic Engineering, Guangdong University of Technology, Guangzhou 510006, China

**Keywords:** carbon nanotubes, laser lift-off, reduced laser threshold, laser ablation

## Abstract

Laser lift-off (LLO) is a critical process for separating ultra-thin polyimide (PI) films in flexible electronics manufacturing, yet traditional methods often induce thermal and mechanical damage due to high laser energy processing. To address this, we propose a low-threshold LLO method by integrating carbon nanotubes (CNTs) at the interface between a 500 nm PI film and a glass substrate. The interfacial thermal dynamics and separation quality were evaluated through finite element simulations and experimental validations using a 355 nm ultraviolet nanosecond laser. Results demonstrate that CNTs significantly enhance interfacial ultraviolet absorption and promote lateral heat diffusion due to their high axial thermal conductivity. This mechanism broadens the thermal decomposition zone and suppresses vertical heat transfer, thereby reducing the required LLO threshold from 180 mJ/cm^2^ to 120 mJ/cm^2^. Furthermore, the integration of CNTs reduces interfacial adhesion and alters the separation dynamics, resulting in the formation of smoother blisters with increased diameters and reduced heights compared to conventional LLO. These effects effectively minimize thermal and mechanical damage to the ultra-thin PI film and its integrated devices. This CNT-assisted LLO approach provides an efficient, low-damage solution for ultra-thin film separation, showing strong potential for advancing high-performance flexible electronics.

## 1. Introduction

Since the initial investigation of laser ablation-driven separation in organic polymers, laser lift-off (LLO) has become a core process for fabricating flexible electronics and photonic chips [1]. It enables the precise delamination of different material layers from a substrate through laser-induced energy transfer. In the post-Moore era, demand for ultra-thin, large-area flexible OLED displays, wearable biosensors, and similar products has fueled the shift toward ultra-thin device architectures. Polyimide (PI) films with a thickness of less than 1 μm are ideal substrates for these devices due to their excellent thermal stability, mechanical flexibility, and chemical inertness. Experimental work further confirms that thinner PI films show improved tensile fatigue life and mechanical reliability—key for conformable electronics [2,3,4]. Efficient separation of ultra-thin PI films from rigid substrates via LLO is critical to ensuring product performance in flexible electronics manufacturing [5]. Its value is reflected in two key aspects. (1) Yield improvement in ultra-thin wafer processing: mature LLO solutions have demonstrated the capability to limit ultra-thin wafer breakage to below 5%. Compared with unprotected processes that typically yield 75–85%, LLO-assisted approaches can elevate the yield to over 95%, substantially reducing material loss and production cost. (2) Enabling next-generation advanced packaging: LLO technology serves as a foundational process for advanced packaging architectures, including 3D integrated circuits, chiplets, and wafer-level packaging. These are essential for the semiconductor industry to overcome performance bottlenecks such as signal delay and power consumption, which conventional planar integration cannot resolve. The strong industrial demand and broad application prospects underscore the urgent need for continued research and development in LLO-related technologies, with particular emphasis on low-damage and high-throughput process optimization. Ultra-thin film presents major challenges for conventional LLO technology: the 500 nm PI film has extremely low mechanical strength, leaving integrated devices prone to failure from photochemical degradation, cumulative thermal conduction, and mechanical stress concentration during laser irradiation. Researchers have explored various solutions to this problem, achieving significant progress [6,7,8,9,10]. For instance, Kim et al. used an α-GaOx sacrificial layer to lower the LLO threshold of a 5 μm thick PI film from 220 mJ/cm^2^ to 150 mJ/cm^2^, reducing thermal stress-induced microcracks [8]. However, sacrificial layers like α-GaOx and SiNx have mismatched coefficients of thermal expansion with PI films and glass substrates, leading to irreversible deformation of ultra-thin films; they also require extra PVD and photolithography steps, increasing process complexity and costs [6,7,8,9,10]. Bian et al. proposed the laser-induced interface separation multi-pass scanning strategy, achieving wrinkle-free delamination of 1 μm thick PI films by scanning five times at 80 mJ/cm^2^—this reduces single thermal shock damage but suffers from long process cycles, strict alignment requirements (±1 μm tolerance), and cumulative thermal deformation, making it unsuitable for large-scale production [11,12,13,14]. These findings offer valuable insights for addressing LLO’s technical bottlenecks: functional interlayers reconfigure laser energy transmission and dissipation through material-specific properties, providing an effective way to optimize interfacial thermal behavior and mechanical characteristics; low-energy directional process design points to the core direction for minimizing device damage [4]. Achieving efficient, damage-free delamination of ultra-thin PI films with low energy density and a single laser scan remains an unresolved core challenge, especially for high-precision flexible electronics manufacturing.

Based on the results gleaned from the aforementioned studies, the present work adopts the design paradigm of functional interlayers to propose a novel LLO methodology, the core of which resides in the integration of CNTs at the interface between the ultra-thin PI film and the glass substrate. The selection of CNTs is primarily motivated by their unique one-dimensional structure and anisotropic thermal conductivity. CNTs provide extremely high axial thermal conductivity, forming an effective lateral heat diffusion channel along the interface that significantly broadens the thermal decomposition zone and prevents excessive vertical heat concentration. Fujii et al. accurately measured the thermal conductivity of a single CNT, confirming its outstanding heat conduction performance [15]. This study provides a theoretical foundation for utilizing CNTs in efficient thermal management. Although metal nanoparticles possess higher UV absorption, their UV absorption relies on plasmon resonance with a narrow absorption band, and they are prone to melting and agglomeration under high-temperature laser irradiation, destabilizing the interface structure. Silicon-based nanoparticles also possess higher UV absorption, but they are highly susceptible to surface oxidation during aqueous pulsed laser ablation preparation; Vaccaro et al. showed that a silica shell layer forms on laser-ablated silicon nanoparticles, substantially weakening their ultraviolet absorption efficiency [16]. Different from the research on graphene-enabled laser lift-off for ultra-thin displays reported by Sumin Kang et al., CNTs exhibit superior comprehensive performance in terms of thermal properties, laser absorption efficiency and practical application costs [17]. CNTs and graphene show distinct differences in thermal properties and laser absorption performance: both have excellent axial thermal conductivity, exceeding 3500 W·m^−1^·K^−1^ [18,19,20]. For laser absorption, graphene displays superior absorption in the ultraviolet region, with absorption efficiency increasing nearly linearly with the number of layers. However, large-area application of graphene depends on high-precision transfer processes, which tend to introduce defects that impair absorption uniformity. Moreover, the absorption efficiency of single-layer graphene is quite low, which requires multi-layer stacking to improve performance and thus increases process complexity. In contrast, CNTs possess a high laser absorption coefficient of ~10^5^ cm^−1^, enabling efficient absorption of ultraviolet and other wavelengths. Importantly, CNTs do not need complex transfer procedures and can be uniformly dispersed via solution spin-coating; their laser absorption uniformity is less affected by the dispersion state, resulting in stronger process compatibility. In addition, CNTs exhibit remarkable cost advantages in the LLO process, making them more suitable for industrialization. Graphene requires complex and high-cost preparation and transfer processes, with the need for multi-layer stacking further driving up costs. In contrast, CNTs can be mass-produced at low cost without transfer procedures, achieving interfacial integration via solution spin-coating; low-concentration dispersions suffice for process requirements, with prominent marginal cost advantages in large-scale production.

To verify the efficacy of CNT integration, this study compares the conventional LLO method and the CNT-assisted LLO method in terms of laser threshold energy and the PI film delamination quality. Concurrently, a two-dimensional finite element model was established to simulate the temperature field distribution. Simulation results demonstrated that this method reduces the LLO threshold by approximately 42%. Experimental validation conducted on 500 nm PI films shows that the incorporation of CNTs decreases the delamination threshold from 180 mJ/cm^2^ (conventional protocol) to 120 mJ/cm^2^. The lateral thermal diffusion effect of CNTs suppresses vertical heat transfer, thereby minimizing thermal damage and mechanical deformation—a conclusion corroborated by comparative focused ion beam scanning electron microscopy (FIB-SEM) analysis of blister morphologies after laser lift-off. This work provides a more efficient, reliable, and cost-effective low-threshold solution for LLO technology, which is expected to accelerate the development of next-generation flexible electronic devices such as high-performance organic light-emitting diodes (OLEDs), wearable devices, and flexible optoelectronic integrated circuits (OEICs). However, within the broader framework of LLO research, a significant gap remains between laboratory proof-of-concept demonstrations and the stringent requirements of industrial-scale production. The key challenges, including the spatial uniformity of the interlayer across large-area substrates, the long-term stability of the interface under repeated thermal and mechanical cycling, and the compatibility with diverse device architectures, remain largely unexplored. Achieving efficient, damage-free delamination of ultra-thin PI films with low energy density and a single laser scan represents an important step toward addressing these challenges, yet it constitutes only the first stage of a broader development pathway.

## 2. Materials and Methods

**Figure 1:**
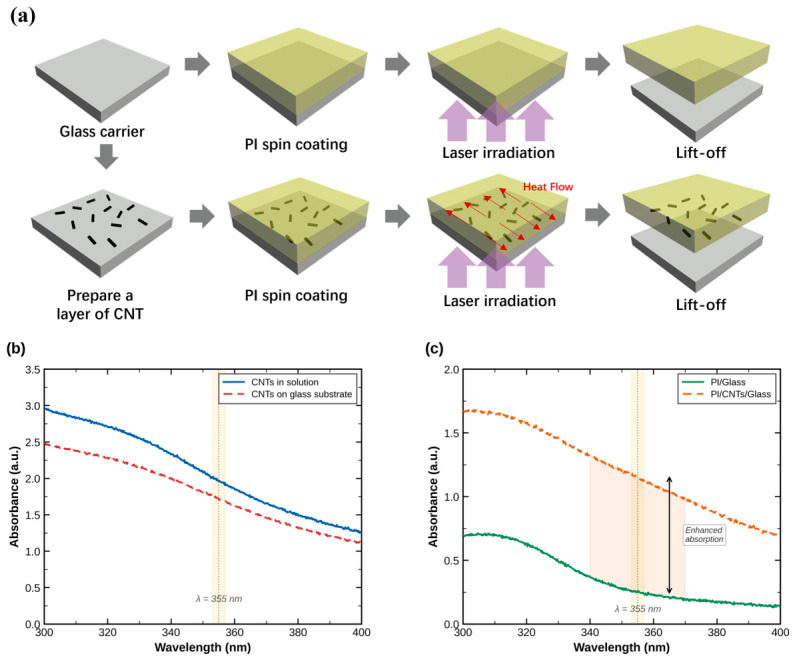
(**a**) The schematic diagrams of the working processes of CNT-LLO and traditional LLO. (**b**) The UV absorption spectrum of the CNT solution and the CNTs on the glass substrate. (**c**) The UV absorption spectrum of the PI/Glass and the PI/CNTs/Glass.

This section details the fundamental working mechanism of the proposed CNT-assisted laser lift-off (LLO) technology, with a specific focus on the comparative process flows illustrated in Figure 1a. The LLO process requires laser irradiation from the backside of the transparent glass substrate to initiate ablation inside the PI film. In the conventional LLO process, the incident laser energy penetrates the glass and is directly absorbed by the pure PI layer. This direct absorption typically induces a vertically concentrated thermal decomposition zone within the PI film, leading to localized overheating, sharp blister formation, and subsequent mechanical damage to the integrated devices. In contrast, the CNT-assisted LLO strategy introduces a functional CNT interlayer between the glass substrate and the ultra-thin PI film. When the backside laser pulse reaches the interface, the CNT layer acts as a highly efficient primary absorption medium. This is because CNTs (Tanfeng Technology, Suzhou, China)can significantly enhance the UV absorption at the interface. The optical absorption of the CNT solution, the spin-coated CNT layer, and the composite structures (PI/CNTs/glass) were systematically evaluated using a UV-Vis-NIR spectrophotometer, as shown in Figure 1b,c. The measurements demonstrated strong absorption of both the CNT solution and the spin-coated CNT coating at the target wavelength of 355 nm. A direct comparison of the composite absorption spectra between the PI/CNTs/glass and PI/glass structures revealed a substantial increase in absorption with CNT integration. This enhanced absorption ensures that a greater proportion of the incident laser energy can be captured precisely at the interface, thereby lowering the overall energy threshold required to trigger the photo-thermal decomposition of the PI film. The exceptional axial thermal conductivity of CNTs establishes rapid lateral heat diffusion pathways, effectively broadening the thermal decomposition zone horizontally while suppressing excessive vertical heat transfer into the PI film. This mechanism changes the interfacial thermal dynamics, protecting the attached optoelectronic devices from thermal degradation and mechanical stress.

The effectiveness of the CNT-LLO process is intrinsically linked to the contrast in thermophysical properties between the CNTs and the PI film. PI (Shenzhen Farcien Applied Materials Co., Ltd., Shenzhen, China)exhibits a relatively low thermal conductivity of approximately 0.1–0.3 W·m^−1^·K^−1^, a density of 1.42 g·cm^−3^, and an absorption coefficient of 2 × 10 cm^−1^. Conversely, CNTs possess an extremely high axial thermal conductivity exceeding 3000 W·m^−1^·K^−1^, which is several orders of magnitude higher than that of PI, alongside a density of roughly 1.3 g·cm^−3^ and a specific heat capacity of about 0.7 J·g^−1^·K^−1^. During ultraviolet laser irradiation, the CNTs act as highly efficient nanoscale heating wires due to their strong UV absorption. The combination of high absorption and exceptional lateral thermal conductivity allows the CNT layer to rapidly convert optical energy into heat and distribute it evenly across the interface, validating the theoretical basis for the reduction in the laser lift-off threshold.

## 3. Model Calculation

To further investigate the specific role of CNTs in the LLO process, we established a two-dimensional finite element photo-thermal model using COMSOL Multiphysics (version 6.3) to investigate the temperature variations at the PI/glass interface due to the highly localized heat transfer from the laser pulse. The LLO process requires laser irradiation from the backside of the glass, which initiates ablation inside the PI film rather than at the interface. The calculation of the temperature distribution in PI is crucial in this model.

The computational domain was discretized using a physics-controlled extremely fine mesh to ensure high resolution at the material interfaces, maintaining a high mesh quality metric throughout the critical heat transfer zones. The simulation utilized a transient solver with a generalized alpha time-stepping method, dynamically adjusting the time steps to capture the rapid thermal transients during the 20 ns laser pulse.

First, the 2D heat diffusion equation is given (with the coordinate fixed at the interface between PI and glass).

(1) $$\rho C_{p}\left(T\right)\frac{\partial T}{\partial t}=\frac{1}{\partial x}\left[K_{x}\left(T\right)\frac{\partial T}{\partial x}\right]+\frac{1}{\partial z}\left[K_{z}\left(T\right)\frac{\partial T}{\partial z}\right]+Q$$

Here, T is the temperature, ρ is the density, C_p_(T) is the specific heat capacity, K(T) is the thermal conductivity, and Q is the source term. The source term Q includes the absorption of the laser (the term on the left) and the term representing the heat loss from chemical reactions. Although the specific heat capacity and thermal conductivity are formally expressed as temperature-dependent functions to represent the general theoretical framework, in the actual numerical implementation within COMSOL, these parameters were approximated as effective constants based on their average values across the relevant temperature range to optimize solving efficiency while preserving the core physical trends.

(2) $$Q=\frac{\alpha I}{\rho C_{p}\left(T\right)}-\frac{∆H_{e}N_{0}\left(1-n_{b}\right)k_{0}\exp\left(-\frac{E_{b}}{k_{B}T}\right)}{\rho C_{p}\left(T\right)}$$

Here, $∆H_{e}$ is the enthalpy required to break a bond, while N_0_ is the number of bonds per unit volume. The distribution of the laser intensity I(x, z, t) in the PI film can be described by the Beer–Lambert law as follows:

(3) $$\frac{\partial I\left(x,z,t\right)}{\partial z}=-I\alpha \left(x,z,t\right)$$

Here, α represents the absorption coefficient of the PI materials, which can be regarded as a constant for nanosecond laser pulses. The boundary condition is considered as I (0, t) = (1 − ζ) I_0_(t), where ζ denotes the proportion of energy loss of the laser pulse after passing through the transparent glass substrate (set to approximately 5%, accounting for reflection and absorption). I_0_(t) is the time-dependent laser intensity, which can be simplified to a rectangular waveform with a pulse width of 20 ns.

**Figure 2:**
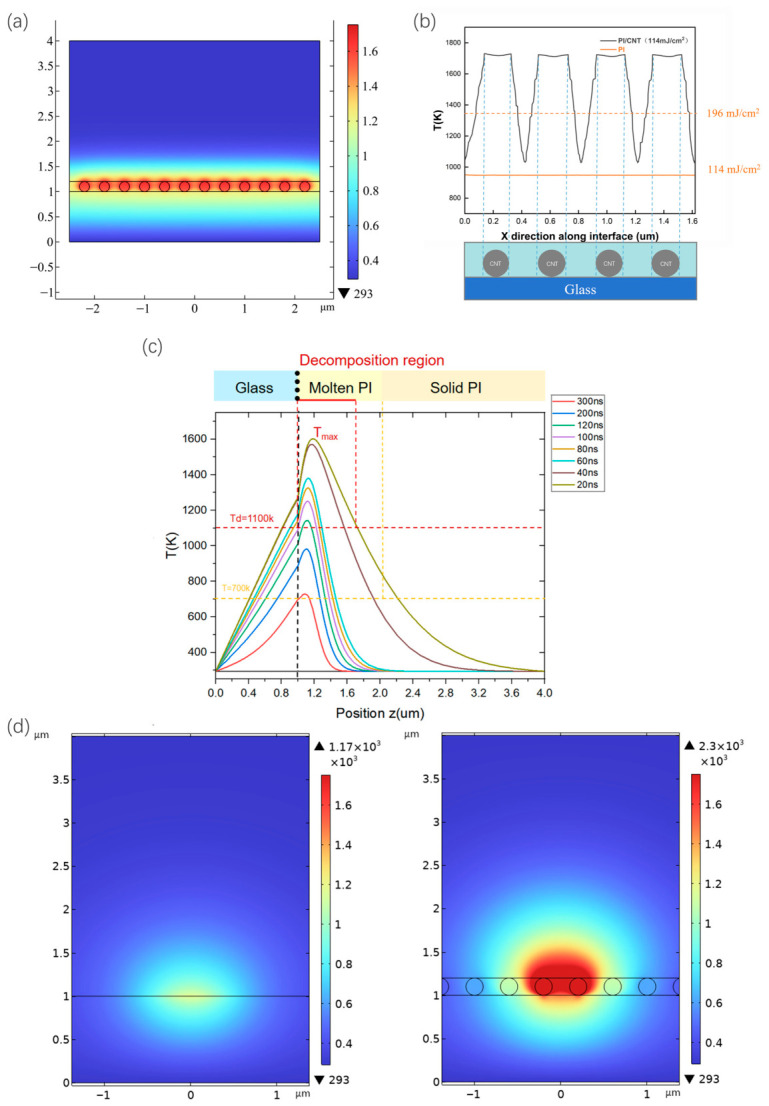
Two-dimensional finite element simulation of the temperature distribution in the PI/CNTs/glass system with a nanosecond laser, illustrating the role of CNTs in enhancing interfacial heat absorption and promoting lateral thermal diffusion. (**a**) Two-dimensional finite element simulation of the temperature distribution in PI/CNTs/glass. (**b**) Comparison of the simulated temperature distribution between the PI/glass (red solid line) and the PI/CNTs/glass (black solid line) after 20 ns, 114 mJ/cm^2^ LLO and the laser threshold to reach the same average temperature of PI/CNTs/glass for pure PI (red dashed line). (**c**) Time-dependent temperature distribution at the PI/glass interface under a single 20 ns LLO of 114 mJ/cm^2^. (**d**) The temperature distribution of the PI films with/without CNTs.

In our model, the CNTs were integrated on the glass surface and embedded in the PI film, as shown in Figure 2a. The radius of CNTs was set as 100 nm with the array period of 400 nm, while the thickness of PI was set as 3 μm to investigate the heat diffusion process. The choice of a 3 μm thickness instead of the actual PI film thickness of 500 nm in the simulation was intentional to provide a sufficiently large computational domain to clearly visualize the vertical heat diffusion profile and the attenuation of the thermal wave before it reaches the upper boundary. PI has a relatively low thermal conductivity (approximately 0.1–0.3 W m^−1^ K^−1^), and the density was set at 1.42 g cm^−3^ with an absorption coefficient of 2 × 10^5^ cm^−1^. CNTs have an extremely high thermal conductivity along the axial direction (>3000 W m^−1^ K^−1^), which is several times that of metals, but their radial thermal conductivity is two orders of magnitude lower. In this model, the axial thermal conductivity is taken as κ∥ = 3500 W m^−1^ K^−1^, the radial thermal conductivity is κ⊥ = 10 W m^−1^ K^−1^, the density is approximately 1.3 g cm^−3^, and the specific heat capacity is approximately 0.7 J g^−1^ K^−1^ [21]. Through the calculation results shown in Figure 2b, it can be found that when the evaporation temperature of polyimide (PI) reaches 1100 K, the laser energy required for traditional LLO technology is as high as 196 mJ/cm^2^. However, for the PI film with CNTs integrated, the laser energy required to reach the same temperature (1100 K) can be significantly reduced to only 114 mJ/cm^2^, due to the achieved high temperature of above 1600 K inside CNTs caused by the high laser absorption of CNTs. This means that the laser energy threshold can be reduced by 42%, indicating that the introduction of CNTs has a significant effect on lowering the energy threshold in the laser separation process. From Figure 2a,b, it can also be found that the interface temperature of PI/CNTs/glass was always higher than that of PI/glass, demonstrating a periodic property between around 1100 K and 1700 K. Along the CNTs laterally, the temperature decreased to the valley of around 1100 K in the middle of two adjacent CNTs. In our model, CNTs can act as powerful heating wires, sending the heat outside to increase the PI temperature and reduce the laser threshold. With the same laser energy, the average temperature at the interface with CNTs inserted was significantly higher than that of pure PI, further proving the high efficiency of CNTs in photo-thermal conversion.

Usually, the laser LLO process of PI is based on the photo-thermal decomposition mechanism of materials within the vertically distributed thermal decomposition zone [22]. Figure 2c shows the time-dependent temperature distribution at the PI/CNTs/glass interface under 20 ns laser irradiation (114 mJ/cm^2^), clearly revealing the ablation mechanism of the traditional LLO method. High temperature can lead to the melting of the layer of PI near the interface since PI completely melts at around 700 K. In addition, after the laser irradiation (20 ns), the maximum temperature of PI is much higher than its thermal decomposition temperature and then decreases rapidly. For the case without CNTs, when laser irradiation occurs, the ablation of PI film mainly takes place in the vertical direction within the film near the interface between the glass and PI, rather than along the interface itself [23,24,25]. However, when CNTs are integrated at the interface, due to their high laser absorption coefficient, they can significantly enhance the absorption of photo-thermal energy at the interface. As a result, we can observe that the maximum temperature point gradually shifts from within the PI film to the interface over time. Moreover, the high horizontal thermal conductivity of CNTs also plays a key role in this process. It can effectively disperse the heat in the horizontal direction at the interface, thereby significantly widening the originally narrow thermal decomposition zone. This widening effect allows PI to stay within the decomposition zone for a longer time, thus improving the efficiency of laser separation.

At the same time, as a direct result of photo-thermal decomposition, gaseous products can be generated within the PI film, which play a crucial role in the separation of the PI film [13]. Usually, the vertically distributed thermal decomposition zone can lead to the initial formation of blisters, and as the gaseous products further expand, the blisters also gradually increase in size. In this process, the inherent strong interfacial adhesion between the glass and the PI film effectively prevents lateral crack propagation to the glass-PI interface. However, the integration of CNTs at the interface can improve the horizontal thermal conduction and reduce the vertical thermal conduction, thereby enlarging the blister size in the lateral direction and improving the LLO efficiency. To demonstrate the diffusion of the heat flow in the lateral direction, the temperature distributions of the PI films with/without CNTs were calculated and compared in Figure 2d after pulse laser irradiation. These two figures of the simulated temperature distributions with/without CNTs integrated can intuitively demonstrate the increase in lateral heat flow through comparison: the left figure without CNTs shows that the high-temperature area after laser irradiation is concentrated in a small local region with a narrow heat diffusion range, indicating weak lateral heat flow, while the right figure with the CNTs exhibits a significant horizontal broadening of the high-temperature area, which reflects a notable increase in lateral heat flow, whereby the horizontal heat transfer capacity is enhanced to transform the high-temperature area from “local concentration” to “lateral dispersion”. This difference in thermal distribution directly confirms the strengthening effect of CNTs on the lateral heat flow.

The simulation results indicate that CNTs can play an important role in the reduction of the laser threshold and the thermal damage to the devices attached to the PI film due to the high laser absorption and lateral thermal conduction.

## 4. Experimental Results

**Figure 3:**
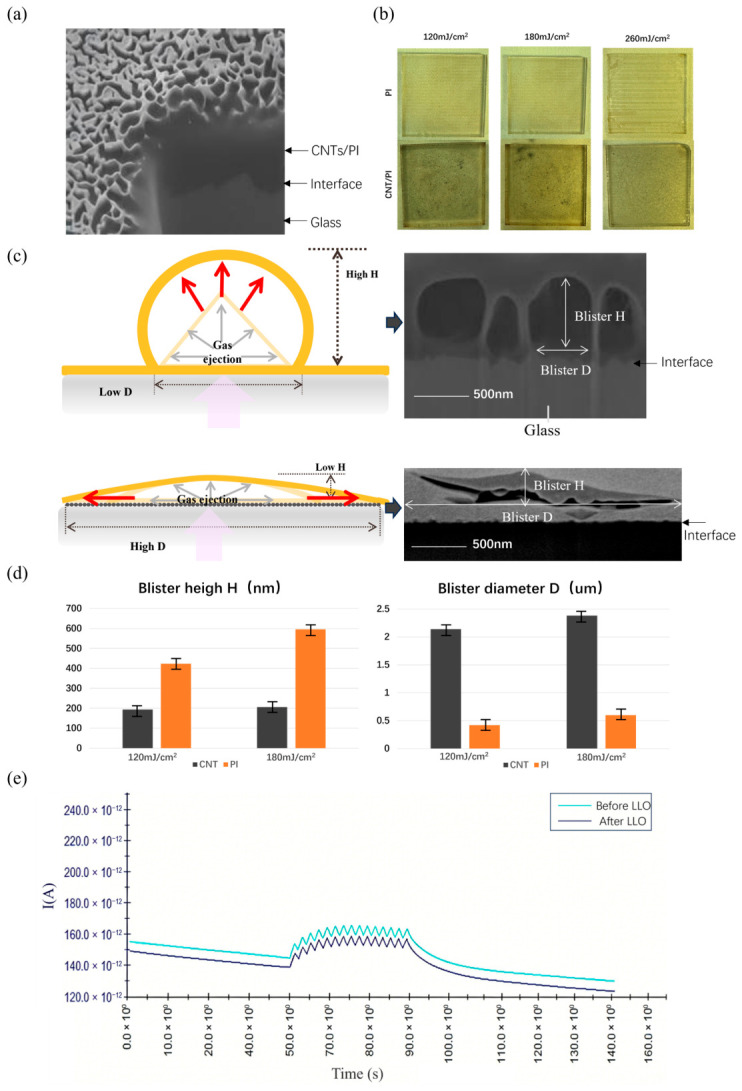
Experimental characterization of the CNT-assisted LLO process, including microstructural analysis of the CNTs/PI composite film, surface morphology comparison after LLO at different laser energy densities, blister shape and height quantification, and functional verification of an integrated optoelectronic synapse device. (**a**) Cross-sectional FIB-SEM image of the non-irradiated CNTs/PI composite film. (**b**) Comparison of sample surfaces after LLO with different laser energies. (**c**) Schematic illustration and vertical cross-sectional FIB-SEM images comparing the blister shapes with and without CNTs after LLO at the threshold energy. (**d**) Comparison of blister heights in samples with and without CNTs at laser energy densities of 120 mJ/cm^2^ and 180 mJ/cm^2^. (**e**) Photocurrent signal of the test optoelectronic synapse device before and after LLO.

To verify the analysis in the above simulation calculations, the CNTs with a size of 200 nm and PI film were spin-coated on the glass substrate for further LLO process. The concentration of CNTs was 0.1 mg/mL. The thickness of the PI film was fixed at 500 nm for studying the separation characteristics of ultra-thin PI films, which are much thinner than those reported in previous LLO studies. Due to the introduction of the CNT layer, the actual thickness of the PI film may vary slightly, but such variation is acceptable within the range of experimental error. To observe the spatial distribution of the CNTs, FIB-SEM and microscopy were performed on the composite film before the LLO. As shown in Figure 3a, the cross-section image illustrates that the PI film attaches closely to the surface of the glass, and the CNTs exhibit a randomly interwoven distribution on the glass surface, as shown in Figure S1 in the Supplementary document. Although the actual spin-coated CNT layer presents this randomly interwoven structure rather than the idealized perfectly aligned geometry assumed in the simulation model, the exceptionally high axial thermal conductivity intrinsic to CNTs ensures that this property remains fully operative within such a configuration. Within this random network, inter-tube contacts establish long-range lateral thermal conduction pathways, enabling the composite layer to produce lateral heat diffusion effects consistent with the simulation results. Despite the apparent orientation contradiction between the perfectly aligned two-dimensional periodic array in the numerical model and the in-plane randomly interwoven network in the experiment, the fundamental physical mechanism remains consistent.

After sample preparation, the LLO process was carried out using a nanosecond laser system in the ultraviolet wavelength range. The laser lift-off process was performed using a fully automated laser debonding system (DSI-S-DB661, Han’s Semiconductor, Shenzhen, China). This system is equipped with an integrated diode-pumped solid-state ultraviolet nanosecond laser source (HSET-355-15, Han’s Semiconductor) operating at a wavelength of 355 nm with an average output power of 15 W. The laser delivers pulses with a duration of 10 to 20 ns and a tunable repetition rate ranging from 10 to 200 kHz, ensuring a high-quality TEM00 spatial mode with a beam quality factor M^2^ of less than 1.3. During the debonding operation, the system provides a maximum scanning speed of 3000 mm/s and maintains a scanning linearity below 0.9 mrad/44° along with a repositioning accuracy of less than 2 μrad. The beam spot uniformity across the irradiated area exceeds 90%, which is critical for achieving a consistent thermal distribution at the interface. (Detailed product specifications can be accessed via the manufacturer’s official website at https://www.szhset.com/productdetail/16.html) (2 March 2026)). The laser lift-off process is conducted using the system with optimized laser parameters tailored for ultra-thin PI-based wafers: a pulse width of 20 ns, forming a near-circular Gaussian beam profile with uniform energy distribution on the sample surface; a max power of 4.5 W; a frequency of 50 KHz; a focal position fixed at 18.2 mm; a scanning speed of 1000 mm/s; and an overlap rate of 50%. The process sequence began with the transfer of ultra-thin PI-based wafers from the storage cassette to the laser processing stage, a step executed with precision to avoid mechanical damage to the fragile PI substrate. Subsequent to the configuration of the aforementioned laser parameters, the galvanometer system performed scanning of the PI layer situated between the glass substrate and the wafer along a programmed path—this scanning strategy was specifically designed to leverage the 50% overlap rate for uniform energy distribution, mitigating localized overheating that could induce PI film wrinkling or carbonization. Then, the wafers were transferred to the substrate separation stage, where a vacuum chuck with adjustable suction pressure was utilized to achieve gentle separation of the glass substrate, which was then stored in a dedicated cassette to prevent contamination. The separated wafers were thereafter conveyed to the cleaning chamber for residual adhesive removal via a mild plasma cleaning process compatible with PI material properties. The cleaned wafers were finally returned to the storage cassette, completing the whole debonding process.

Figure 3b illustrates the comparative LLO experimental results between the PI/CNTs/glass and PI/glass samples at laser energy densities of 120, 180, and 260 mJ/cm^2^. The PI film integrated with CNTs achieved successful lift-off across all tested energy conditions, leaving no significant plastic deformation. In contrast, the conventional PI/glass samples exhibited a narrow laser energy operational range. In conventional LLO, the process window for wrinkle-free lift-off is inherently narrow [6]. At a low energy density of 120 mJ/cm^2^, the PI film only partially separated and failed to detach completely from the glass substrate. When exposed to higher laser energy of 180 and 260 mJ/cm^2^, the PI film could be lifted off but suffered severe wrinkles induced by plastic deformation, posing a critical risk of mechanical damage to any attached electronic devices (please find more microscope images of the samples after LLO in Figure S2 in the Supplementary document). These comparative results rigorously demonstrate that CNT integration substantially broadens the LLO process window. The CNT layer effectively lowers the required laser energy threshold while simultaneously mitigating thermo-mechanical damage, validating the heat dissipation mechanisms predicted by our simulation results.

To further investigate the role of CNTs during the LLO process, the more detailed microstructural characterization in the vertical cross-sectional direction is provided in Figure 3c, which presents FIB-SEM cross-sectional images of the blister morphologies formed after the conventional LLO and CNT-assisted LLO. The flat, horizontal layer visible at the bottom is the glass, and the arched dome represents the blistered region caused by the expansion of gaseous ablation products. The concentration of CNTs and the laser energy were controlled at 0.01 mg/mL and 120 mJ/cm^2^, respectively. Traditional LLO usually utilizes the gas pressure generated by these blisters to help separate the material. From the comparison of the blister shapes after LLO, it can be found that the blisters in traditional LLO are typically sharp and have a relatively high height, which can cause significant mechanical strain and potential damage to the devices on the PI surface. In contrast, the LLO process with CNTs integrated allows for lateral heat diffusion through a horizontally distributed pyrolysis zone, making the initially generated blisters smoother due to the high thermal conductivity of the CNT layer. The differences in blister height and diameter between samples with and without CNTs at laser energy densities of 120 mJ/cm^2^ and 180 mJ/cm^2^ are shown in Figure 3d. To ensure statistical reliability, more than 20 blisters were analyzed for each experimental condition. Figure 3d presents the quantitative characterization of blister height and diameter for both CNTs/PI composite films and pure PI films under two laser fluence conditions of 120 mJ/cm^2^ and 180 mJ/cm^2^, with error bars indicating the standard deviation of multiple measurements. With a laser fluence of 120 mJ/cm^2^, the PI film formed blisters with a mean height of approximately 420 nm and a standard deviation of ±20 nm, whereas the CNTs/PI composite film exhibited a blister with a height of only about 190 nm with a standard deviation of ±25 nm, making the former nearly 2.2 times larger than that of the latter. As the laser fluence increased to 180 mJ/cm^2^, the blister height of the PI film further increased to approximately 590 nm with a standard deviation of ±15 nm, while the composite film remained relatively stable at about 210 nm with a standard deviation of ±25 nm. This indicates a markedly suppressed height effect in the CNTs/PI composite film with increasing laser fluence, and the difference between samples with and without CNTs continued to grow. In contrast to the height variation trend, the CNTs/PI composite film can generate blisters with larger diameters. With 120 mJ/cm^2^ and 180 mJ/cm^2^, the blister diameters of the CNTs/PI composite film were approximately 2.1 μm and 2.35 μm, respectively, both with a standard deviation of around ±0.05 μm, whereas the pure PI film exhibited diameters of only about 0.35 μm and 0.65 μm, respectively, both with a standard deviation of around ±0.08 μm. This indicates that with the integration of the CNT layer, the reduction in blister height and the increase in blister diameter confirm the effect of the CNT layer on blister smoothing. This behavior is attributed to the enhanced UV absorption at the CNT layer, which increases the maximum temperature driven by photo-thermal energy under the same laser energy conditions. The spin-coated CNT network acts as a physical spacer, which significantly reduces the direct contact area between the PI polymer chains and the glass substrate. This physical separation disrupts the formation of strong van der Waals forces and potential hydrogen bonds, which typically dominate the strong interfacial adhesion in conventional PI and glass systems [17]. Consequently, the reduced adhesion energy facilitates the lateral propagation of gaseous ablation products along the interface. This lateral crack growth promotes the formation of wider and smoother blisters. The resulting blisters exhibit a low height and a large diameter, as shown in Figure 3d. Such morphological characteristics effectively reduce the mechanical strain during the laser lift-off process and minimize mechanical damage to the devices attached to the polyimide surface.

To provide a more comprehensive statistical evaluation of the blister morphology, the blister density per unit area was also quantitatively analyzed. We performed a quantitative statistical analysis of the blister density within a representative 3 μm × 3 μm area for both the conventional PI/glass and the CNT-assisted PI/CNTs/glass samples at the threshold energy of 120 mJ/cm^2^. Based on our measurements, the conventional PI/glass samples exhibit a high density of sharp, localized blisters, with an average count of approximately 20–25 blisters per area. In contrast, the integration of the CNT layer significantly alters the blister morphology and distribution. Due to the enhanced lateral heat diffusion and reduced interfacial adhesion provided by the CNTs, the gaseous products propagate laterally, leading to the coalescence of adjacent micro-blisters into larger, flatter domes. Consequently, the blister density in the CNT-assisted samples drastically decreases to approximately 2–3 blisters per area.

To verify the stability of the device during the laser debonding of the PI film with CNTs, after bonding a test optoelectronic synapse device to the surface of the PI film, a comparative analysis was conducted on the photocurrent signal of the device before and after laser debonding, as shown in Figure 3e. The results revealed that the device remained fully functional, and there was a minor degradation in the peak photocurrent signal—approximately a 5–8% decrease compared to the device before LLO. No spontaneous detachment of the PI film from the substrate was observed throughout the entire device fabrication process, including PI coating, CNT integration, and chip bonding. Collectively, these experimental results demonstrate that the introduction of CNTs does not cause substantial degradation to the interfacial bonding stability between the PI film, the device, and the substrate. While it may lead to minor variations in interfacial adhesion, it completely mitigates the risk of premature PI film debonding in subsequent processes and does not compromise the stability or performance of the original devices.

The CNT layer plays a crucial role in the LLO process, mainly due to three key effects: (1) significant enhancement of ultraviolet light absorption at the interface, (2) lateral diffusion of heat, and (3) significant reduction of adhesion at the interface. The experimental results clearly demonstrate the critical role of the CNT layer in the LLO process. The integration of CNTs successfully enabled the delamination of a 500 nm thick PI film from the glass substrate. During this process, the PI film exhibited no observable plastic deformation, and the amount of carbonaceous residue on the glass substrate was notably minimized. These empirical findings directly corroborate our simulation models, confirming that the CNT interlayer can enhance the interfacial ultraviolet absorption and facilitate rapid lateral heat diffusion. Consequently, the localized thermal accumulation and mechanical stress are effectively mitigated. By precisely controlling ultraviolet laser irradiation on the CNTs/PI/glass, a low-damage LLO process can be reliably achieved with lower laser energy densities.

## 5. Conclusions

In this study, we proposed and demonstrated a highly efficient CNT-assisted laser LLO strategy to address the critical challenges of thermal and mechanical damage during the delamination of ultra-thin PI films. Both finite element simulations and experimental validations confirmed that the integration of a CNT interlayer significantly optimizes the interfacial photo-thermal dynamics. The required laser energy threshold for the successful lift-off of a 500 nm PI film was substantially reduced from 180 mJ/cm^2^ to 120 mJ/cm^2^, representing a 33% decrease. This remarkable improvement is achieved by the synergistic effects of the CNTs: enhanced interfacial UV absorption, rapid lateral heat diffusion, and reduced van der Waals adhesion. Consequently, the conventional sharp, high-aspect-ratio blisters were transformed into smooth, wide blisters, effectively mitigating localized mechanical strain and preventing plastic deformation of the ultra-thin substrate. Furthermore, the stable performance of the transferred optoelectronic synapse devices verified that this low-threshold LLO process ensures high interfacial reliability without compromising the device function. By providing a robust, cost-effective, and low-damage delamination method, the CNT-assisted LLO technology paves a promising pathway for the scalable manufacturing of next-generation ultra-thin flexible electronics, including high-performance wearable sensors and large-area flexible displays.

Even though these findings prove the fundamental feasibility of the CNT-assisted LLO mechanism at the laboratory scale, the broader claims regarding its immediate applicability to large-area flexible displays and complex wearable systems still require further empirical substantiation. To bridge the gap between the current proof-of-concept and actual industrial commercialization, several critical follow-up studies can be conducted. First, the scalability of the spin-coated CNT layer can be rigorously evaluated across large-area substrates to ensure the spatial uniformity of the LLO process window. Second, comprehensive reliability testing is required to assess the long-term stability of the CNT interlayer under repeated thermal and mechanical cycling, particularly its compatibility with diverse device architectures and organic semiconductor materials, which may be sensitive to residual carbon. Finally, future research should explore the integration of this CNT-assisted strategy with emerging laser sources, such as femtosecond lasers, to further refine the precision of the delamination process. By addressing these practical challenges, subsequent studies may fully unlock the potential of this technology for advanced flexible electronics manufacturing.

## Data Availability

The original contributions presented in this study are included in the article/Supplementary Materials. Further inquiries can be directed to the corresponding author.

## Supplementary Materials

The following supporting information can be downloaded at: https://www.mdpi.com/article/10.3390/nano16090527/s1, Figure S1: Surface microscopic image of the non-irradiated CNTs on the glass substrate. The image reveals that the spin-coated CNTs exhibit a randomly interwoven distribution, forming a two-dimensional network structure at the interface prior to laser irradiation; Figure S2: Comparison of surface microscopic morphologies between PI and CNTs/PI samples after laser lift-off under various laser fluences. At lower fluences (120 mJ/cm^2^), the pure PI samples show no obvious lift-off marks and are incompletely delaminated, whereas the CNTs/PI samples exhibit distinct lift-off features and achieve significant delamination. At a higher fluence (260 mJ/cm^2^), the surface of the pure PI samples displays signs of overexposure with uneven laser spot distribution, while the surface of the CNTs/PI samples remains uniform without any overexposure phenomenon.

## References

[1] Srinivasan R., Mayne-Banton V. (1982). Self-developing photoetching of poly (ethylene terephthalate) films by far-ultraviolet excimer laser radiation. Appl. Phys. Lett..

[2] Kim Y., Noh Y., Park S., Kim B.-K., Kim H.J. (2020). Ablation of polyimide thin-film on carrier glass using 355 nm and 37 ns laser pulses. Int. J. Heat Mass Transf..

[3] Yan Z., Zhang F., Liu F., Han M., Ou D., Liu Y., Lin Q., Guo X., Fu H., Xie Z. (2016). Mechanical assembly of complex, 3D mesostructures from releasable multilayers of advanced materials. Sci. Adv..

[4] Jang H.W., Kim S.K., Yoon S.M. (2019). Impact of polyimide film thickness for improving the mechanical robustness of stretchable InGaZnO thin-film transistors prepared on wavy-dimensional elastomer substrates. ACS Appl. Mater. Interfaces.

[5] Song H., Luo G., Ji Z., Bo R., Xue Z., Yan D., Zhang F., Bai K., Liu J., Cheng X. (2022). Highly-integrated, miniaturized, stretchable electronic systems based on stacked multilayer network materials. Sci. Adv..

[6] Bian J., Zhou L., Yang B., Yin Z., Huang Y. (2020). Theoretical and experimental studies of laser lift-off of nonwrinkled ultrathin polyimide film for flexible electronics. Appl. Surf. Sci..

[7] Park C.I., Seong M., Kim M.A., Kim D., Jung H., Cho M., Kang I. (2018). World’s First Large Size 77-Inch Transparent Flexible OLED Display. J. Soc. Inf. Disp..

[8] Kim K., Kim S.Y., Lee J.L. (2014). Flexible organic light-emitting diodes using a laser lift-off method. J. Mater. Chem. C.

[9] Zhu C., Guo D., Ye D., Jiang S., Huang Y. (2020). Flexible PZT-integrated, bilateral sensors via transfer-free laser lift-off for multimodal measurements. ACS Appl. Mater. Interfaces.

[10] Bian J., Zhou L.B.Y., Wan X.D., Liu M., Zhu C., Huang Y., Yin Z. (2019). Experimental study of laser lift-off of ultra-thin polyimide film for flexible electronics. Sci. China Technol. Sci..

[11] Kim Y., Park S., Kim B.K., Park W.-J., Kim H.J. (2021). Laser lift-off of polyimide thin-film from glass carrier using DPSS laser pulses of top-hat square profiles. Opt. Laser Technol..

[12] Bian J., Chen F., Ling H., Sun N., Hu J., Huang Y. (2022). Experimental and modeling study of controllable laser lift-off via low-fluence multiscanning of polyimide-substrate interface. Int. J. Heat Mass Transf..

[13] Liu Z., Ling Q., Cai Y., Xu L., Su J., Yu K., Wu X., Xu J., Hu B., Wang X. (2022). Synthesis of carbon-based nanomaterials and their application in pollution management. Nanoscale Adv..

[14] Welin E.R., Le C., Arias-Rotondo D.M., McCusker J.K., MacMillan D.W.C. (2021). Photosensitized, energy transfer-mediated organometallic catalysis through electronically excited nickel (II). Science.

[15] Fujii M., Zhang X., Xie H., Ago H., Takahashi K., Ikuta T., Abe H., Shimizu T. (2005). Measuring the Thermal Conductivity of a Single Carbon Nanotube. Phys. Rev. Lett..

[16] Vaccaro L., Popescu R., Messina F., Camarda P., Schneider R., Gerthsen D., Gelardi F.M., Cannas M. (2016). Self-limiting and complete oxidation of silicon nanostructures produced by laser ablation in water. J. Appl. Phys..

[17] Kang S., Chang J., Lim J., Kim D.J., Kim T.S., Choi K.C., Lee J.H., Kim S. (2024). Graphene-enabled laser lift-off for ultrathin displays. Nat. Commun..

[18] Schäffer S., Reich S., Heunoske D., Lueck M., Wolfrum J., Osterholz J. (2024). Laser-induced decomposition and mechanical degradation of carbon fiber-reinforced polymer subjected to a high-energy laser with continuous wave power up to 120 kW. J. Compos. Sci..

[19] Nan P., Shen Z., Han B., Ni X. (2019). The influences of laminated structure on the ablation characteristics of carbon fiber composites under CW laser irradiation. Opt. Laser Technol..

[20] Liu K., Sun Y., Zhou R., Zhu H., Wang J., Liu L., Fan S., Jiang K. (2009). Carbon nanotube yarns with high tensile strength made by a twisting and shrinking method. Nanotechnology.

[21] Stallard J.C., Tan W., Smail F.R., Gspann T.S., Boies A.M., Fleck N.A. (2018). The mechanical and electrical properties of direct-spun carbon nanotube mats. Extrem. Mech. Lett..

[22] Wei Y., Lin X., Jiang K., Liu P., Li Q., Fan S. (2013). Thermoacoustic chips with carbon nanotube thin yarn arrays. Nano Lett..

[23] Sedao X., Abou Saleh A., Rudenko A., Douillard T., Esnouf C., Reynaud S., Maurice C., Pigeon F., Garrelie F., Colombier J.-P. (2018). Self-arranged Periodic Nanovoids by Ultrafast Laser-induced Near-field Enhancement. ACS Photonics.

[24] Wang F., Liu Q., Xia J., Huang M., Wang X., Dai W., Zhang G., Yu D., Li J., Sun R. (2023). Laser lift-off technologies for ultra-thin emerging electronics: Mechanisms, applications, and progress. Adv. Mater. Technol..

[25] Feng H., An D., Tu H., Bu W., Wang W., Zhang Y., Zhang H., Meng X., Wei W., Gao B. (2020). A passive video-rate terahertz human body imager with real-time calibration for security applications. Appl. Phys. B.

