# Supplementary material for: Carbon-Nanotube-Enabled Low-Threshold Laser Lift-Off for Ultra-Thin Polyimide Films
Source: Nanomaterials (Basel). 2026 Apr 27;16(9):527. doi: 10.3390/nano16090527 (PMC13165120; doi:10.3390/nano16090527)
Supplement: Supplementary file 1 [file nanomaterials-16-00527-s001.zip › nanomaterials-4226697-supplementary.pdf]

Supplementary documents

Carbon nanotube enabled low-threshold laser lift off for ultra-thin polyimide films

Junwei Fu [1,2,†], Yachong Xu [1,2,†], Run Bai [1,2], Zhenzhen Sun [1,2], Yili Zhang [1,2], Rui Yang [3], Zijuan Han [4], Fanfan Wang [3] and Boyuan Cai [1,2,*]

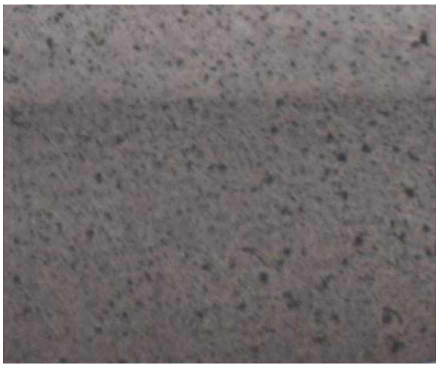

Figure S1. Surface microscopic image of the non-irradiated CNTs on the glass substrate. The image reveals that the spin-coated CNTs exhibit a randomly interwoven distribution, forming a two-dimensional network structure at the interface prior to laser irradiation.

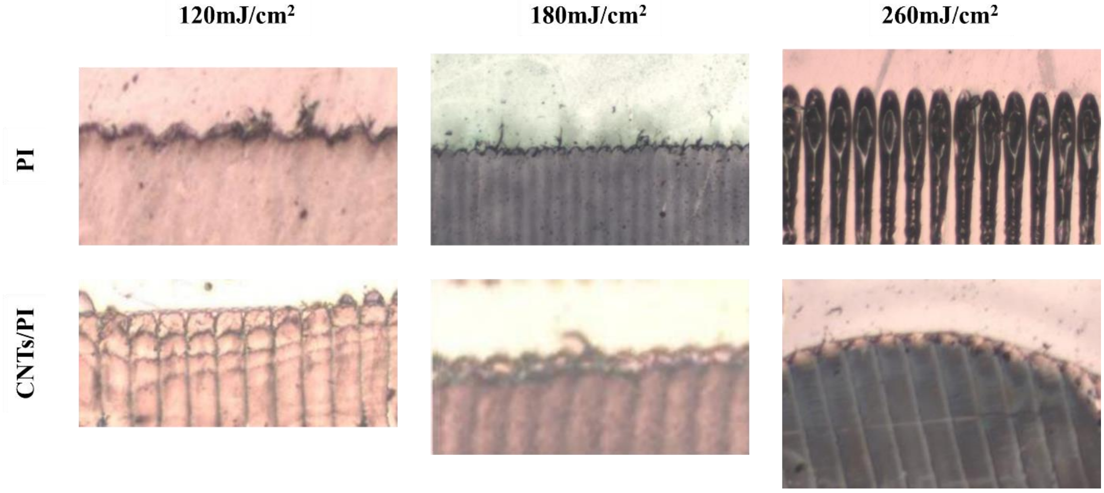

Scheme S2. Comparison of surface microscopic morphologies between PI and CNTs/PI samples after laser lift-off under various laser fluences. At lower fluences (120 mJ/cm^2), the pure PI samples show no obvious lift-off marks and are incompletely delaminated, whereas the CNTs/PI samples exhibit distinct lift-off features and achieve significant delamination. At a higher fluence (260 mJ/cm^2), the surface of the pure PI samples displays signs of overexposure with uneven laser spot distribution, while the surface of the CNTs/PI samples remains uniform without any overexposure phenomenon.
